# In Situ TEM Study of Microstructure Evolution of Zr-Nb-Fe Alloy Irradiated by 800 keV Kr^2+^ Ions

**DOI:** 10.3390/ma10040437

**Published:** 2017-04-22

**Authors:** Penghui Lei, Guang Ran, Chenwei Liu, Chao Ye, Dong Lv, Jianxin Lin, Yizhen Wu, Jiangkun Xu

**Affiliations:** College of Energy, Xiamen University, Xiamen 361102, Fujian, China; p.h.lei@foxmail.com (P.L.); asucba@163.com (C.L.); kim.yc@foxmail.com (C.Y.); dong.lv@foxmail.com (D.L.); jxlin@stu.xmu.edu.cn (J.L.); yzwxiyang@163.com (Y.W.); jiangkunxu@163.com (J.X.)

**Keywords:** ion irradiation, in situ TEM, zirconium alloy, nanoparticle, nanocrystallization

## Abstract

The microstructure evolution of Zr-1.1Nb-1.51Fe-0.26Cu-0.72Ni zirconium alloy, irradiated by 800 keV Kr^2+^ ions at 585 K using the IVEM-Tandem Facility at Argonne National Laboratory, was observed by in situ transmission electron microscopy. A number of β-Nb precipitates with a body-centered cubic (BCC) structure were distributed in the as-received zirconium alloy with micrometer-size grains. Kr^2+^ ion irradiation induced the growth of β-Nb precipitates, which could be attributed to the segregation of the dissolved niobium atoms in the zirconium lattice and the migration to the existing precipitates. The size of precipitates was increased with increasing Kr^2+^ ion fluence. During Kr^2+^ iron irradiation, the zirconium crystals without Nb precipitates tended to transform to the nanocrystals, which was not observed in the zirconium crystals with Nb nanoparticles. The existing Nb nanoparticles were the key factor that constrained the nanocrystallization of zirconium crystals. The thickness of the formed Zr-nanocrystal layer was about 300 nm, which was consistent with the depth of Kr^2+^ iron irradiation. The mechanism of the precipitate growth and the formation of zirconium nanocrystal was analyzed and discussed.

## 1. Introduction

Zirconium alloys are used as fuel cladding tubes and structural materials in water-cooled nuclear reactors due to their low thermal neutron absorption cross section, excellent resistance to corrosion, remarkable resistance to irradiation-induced swelling and creep, and adequate mechanical properties [1]. Along with increased fuel burnup and extended reload cycle of fuel assembly, zero tolerance on nuclear accidents requires further improvement of the properties and performance of zirconium alloy fuel cladding. In recent years, various new zirconium alloys have been designed and developed in China, USA and Europe [2]. In the recent accident tolerant fuel (ATF) project, new types of zirconium alloys with better comprehensive properties and performance are called as safer fuel cladding materials [3]. One of the most important approaches used in improving properties and performance of zirconium alloys is the changing concentrations of alloying elements, such as Nb, Sn, Cu, Fe, etc. [4]. N36 zirconium alloy with Zr-1Sn-1Nb-0.3Fe was developed in China. The material shows a higher performance than Zr-4 alloy [5]. In order to further improve comprehensive performance of zirconium alloys, a new type of zirconium alloy with the composition of Zr-1.1Nb-1.51Fe-0.26Cu-0.72Ni is developed in the present work.

In service, zirconium alloys will be subjected to not only X-rays and neutrons, but also significant ion fluxes that vary in type, nature, energy, and intensity. Therefore, it is important to investigate the irradiation behavior of zirconium alloys, especially for a new zirconium alloy before using it in commercial nuclear reactors. The irradiation with high energetic particles will induce the degradation of properties and performance including irradiation hardening, embrittlement, and swelling. These irradiation-induced damages originate from the microstructure changes such as amorphization, nanocrystallization, particle dissolution or precipitation, formation of dislocation, and voids. Knowledge of the microstructure evolution under irradiation is important for predicting in-service behaviors of zirconium alloy components.

In the past several decades, the irradiation behaviors of zirconium alloys have been widely investigated [5,6,7,8,9,10,11,12,13]. The main investigations conducted so far including the following: (i) phase changes, nanocrystallization, and amorphization [5,6]; (ii) particle dissolution or precipitation [7]; (iii) the effect of chemical composition and preparation technology on the irradiation behaviors [8,9]; (iv) helium behaviors in zirconium alloys [10]; (v) dislocation formation and characteristic [11]; (vi) post-irradiation corrosion behaviors [12]; (vii) computer simulation such as molecular dynamics for irradiation behaviors [13]. In addition, the microhardness obtained by nanoindenter is an important method for characterizing the mechanical properties before and after ion irradiation because of the limited irradiation depth. The nanoindentation is widely used in thin film metallic glass [14,15] and ion irradiated samples [16]. However, there are still some questions that need to be addressed and answered, such as the irradiation-induced growth behavior of precipitates, the amorphization mechanism of zirconium crystals.

The new type of zirconium alloy with the composition of Zr-1.1Nb-1.51Fe-0.26Cu-0.72Ni is developed on the basis of N36 zirconium alloy by means of adding the chemical content of elemental Ni, Fe, and Cu, and eliminating elemental Sn. Elemental Cu and Fe can increase mechanical strength and corrosion resistance [17]. Elemental Nb can enhance corrosion and irradiation-induced swelling resistance [17,18,19]. Although elemental Sn can improve creep resistance, it decreases corrosion resistance [18]. Liu [20,21] reported a zirconium alloy with uniform distribution of fine β-Nb particles containing iron element had high corrosion resistance. Nb atoms were easily dissolved into zirconium lattice, but the formation of precipitates containing Nb would reduce the amount of Nb presenting as a solid solution in the α-Zr crystal structure [22]. Irradiation is likely to accelerate precipitate dissolution or coarsening, prompt phase transformation, and induce new phase formation [7]. The redistribution of microalloying elements was observed under irradiation. Perovic [23] reported that ion irradiation induced the redistribution of alloy elements, the desolvation of elemental Nb from β-Nb precipitates, and the increase in solid solution ability of alloying elements in zirconium matrix. Shishov [24,25] presented a similar result of Zr-Nb-Sn-Fe alloys. Meanwhile, during neutron irradiation, the elemental Fe depleted from β-Nb precipitates and some precipitates enriched with Nb atoms were formed. However, Sylvie Doriot [26] reported the diameter of β-Nb particles increased with irradiation dose, while their number density did not exhibit a significant decrease. For the highest dose (13.1 × 10^25^ n/m^2^), the measured particle density in M5 appeared to decrease. Kai [27] reported the dissolution of ZrFe_2_ precipitates, the transformation of Zr(Fe,Cr)_2_ precipitates from hcp to fcc structure, and the nucleation and growth of Zr_4_Sn precipitates in a Zr-4 alloy irradiated by proton with ion dose up to 1 dpa at 623 K. The different research results in Ref. [23,24,25,26,27] could be due to the irradiation experiment conditions (such as irradiated by 3.58 × 10^24^ n/m^2^ at 583 K for 52 days in-reactor [23], under BOR-60 irradiation with *E* > 0.1 MeV at different temperature [24] and 1 MeV H^+^ irradiation at 350 °C up to a total dose of 1 dpa [27]) and the chemical compositions (such as Zr-2.5%Nb [23], upgraded Zr-1%Nb and Zr-1%Nb-0.35%Fe-1.2%Sn [25], and Zr-1.53%Sn-0.22%Fe-0.11%Cr [27]). However, some scientific questions related to the irradiation behaviors of precipitates in zirconium alloys needed to be further investigated.

Moreover, most of the irradiation experiments and subsequent microstructure analysis in the previous studies were carried out in separate steps and did not reflect irradiation behaviors of zirconium alloys in real time and space. In situ transmission electron microscopy (TEM) observation under ion irradiation is a very useful technique to study mechanisms of damage accumulation and reveal the progressive microstructure changes, which is not only important for understanding the fundamentals of irradiation damage but also important for obtaining irradiation damage data in real time and space to establish basic irradiation rules that can be used to estimate in-service irradiation life of a new zirconium alloy with Zr-1.1Nb-1.51Fe-0.26Cu-0.72Ni. Although the physical mechanism of ion irradiation is different with that of neutron irradiation, the same microstructural characteristics such as the distribution and size of dislocation loops and voids in the matrix, and the chemical composition segregation at the grain boundary, will be obtained during ion irradiation [28,29]. Meanwhile, there is a test standard ASTM E521-1996 (Reapproved 2009) “Standard practice for neutron radiation damage simulation by charged-particle irradiation” [30]. Therefore, it is effective to use ion irradiation to simulate neutron irradiation effect.

To understand the microstructure evolution and precipitate behaviors of Zr-1.1Nb-1.51Fe-0.26Cu-0.72Ni zirconium alloy during krypton ion irradiation, an in situ irradiation experiment was carried out in the IVEM-Tandem facility. In this study, an 800 keV Kr^2+^ ion beam was used to (1) simulate heavy particle irradiation effect; and (2) simulate gas bubble behaviors. The mechanisms of precipitate evolution and the nanocrystallization of zirconium crystal were analyzed and discussed.

## 2. Experimental Methods

The zirconium alloy with Zr-1.1Nb-1.51Fe-0.26Cu-0.72Ni (wt %) chemical composition, which came from a fuel cladding tube, was used in the present work. The sheet sample of 10 × 3 × 0.5 mm^3^ in dimensions was first cut from as-received cladding tube by a precision diamond knife-cutting machine and then ground to less than 100 μm in thickness by SiC sandpaper. After that, the samples with 3 mm in diameter were obtained by a hole-punching machine. Both sides of each sample were ground using SiC sandpaper from 240 to 5000 grit and then polished using 1 μm diamond paste. After thinning the thickness to about 30 μm, the samples were twin-jet electropolished using a mixed solution with 60 mL of HClO_4_, 350 mL of 2-butoxyethanaol, and 590 mL of methanol to perforation for TEM observations. After twin-jet electropolishing, the thickness of TEM sample with 3 mm in diameter was about 100 nm near the perforation position and was about 30 μm near the edge of TEM sample. The sample thickness continuously changed from 30 μm near the edge to 100 nm near the perforation position. Therefore, during ion irradiation, the region monitored by in situ TEM was located near the perforation position (about 100 nm in thickness). After ion irradiation, the region for preparing the cross-sectional specimen in the ion-irradiated TEM sample was located near the edge (about 30 μm thickness). Ex situ TEM and HRTEM were carried out in a JEM 3011 electron microscope (JEOL, Tokyo, Japan) with 300 keV of electron voltage.

Ion irradiations were conducted at 585 K with 800 keV Kr^2+^ using the IVEM-Tandem Facility (National Electrostatics Corp., Middleton, WI, USA) at Argonne National Laboratory, USA. The sample temperature was provided and controlled by a Gatan TEM sample holder. The facility consists of a modified H-9000 electron microscope that is interfaced to a 650 KV NEC ion accelerator. The microstructure evolution was monitored in situ during ion irradiation using a 300 keV electron beam.

**Figure 1:**
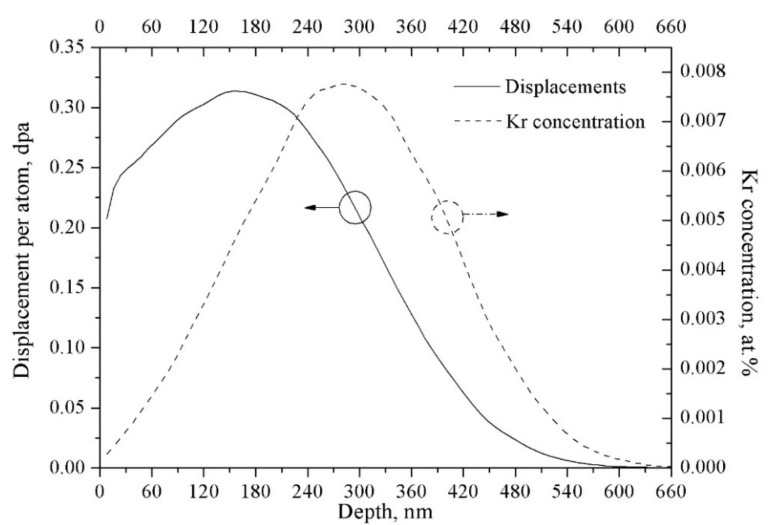
Depth profile of displacement damage and implanted ion concentration in zirconium alloy irradiated by 800 keV Kr^2+^ to 1.0 × 10^18^ ions/m^2^ calculated by SRIM 2008 (Quick mode).

The Kr-ion flux was kept at 6.25 × 10^15^ Kr^2+^ ions/m^2^·s to prevent excessive beam heating. The depth distribution of implanted Kr^2+^ ions and displacement damage was simulated using the Stopping and Range of Ions in Matter (SRIM) code in quick Kinchin-Pease mode (SRIM.EXE, (C) 1984–2013, James F. Ziegler, Annapolis, MD, USA). The atom displacement threshold energy (*E_d_*) of zirconium was designed to be 40 eV. The SRIM simulation of 800 keV Kr^2+^ into zirconium alloy indicated a peak value of 0.0078 atom % Kr ion concentration at a 280 nm depth after irradiation with 1.0 × 10^18^ Kr^2+^ ions/m^2^ fluence (equivalent to 1.0 Kr^2+^/nm^2^), as indicated by the dash line in Figure 1. The peak displacement damage at a 160 nm depth was 0.313 dpa after irradiation with 1.0 × 10^18^ Kr^2+^ ions/m^2^ fluence, as indicated by the full line in Figure 1. Multiplying these value by the implanted ion fluence gave the peak atomic density of implanted Kr and the peak displacement damage. In the present work, the total Kr^2+^ ion fluence was set as 1.7 × 10^20^ Kr^2+^ ions/m^2^, so the calculated peak value of displacement damage and Kr^2+^ ion concentration in Zr alloy were 53 dpa and 1.33 atom %, respectively. The displacement damage of zirconium alloy fuel cladding is approximately 10 dpa at water-cooled nuclear reactors. Along with increased fuel burnup and extended reload cycle of fuel assembly, and zero tolerance on nuclear accidents, zirconium alloy fuel cladding needs to be kept a stable ability at high displacement damage, even under a severe nuclear accident. Therefore, in the present work, the high displacement damage (53 dpa) was designed and carried out for this new type of zirconium alloy.

## 3. Results and Discussion

### 3.1. Microstructure Analysis of As-Received Alloy

**Figure 2:**
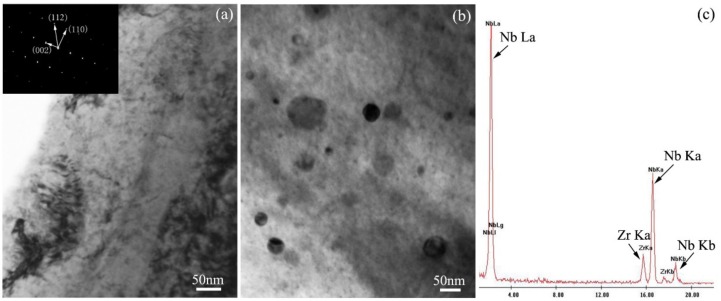
Bright field TEM images showing the as-received zirconium alloy, (**a**) zirconium crystal without precipitates. (**b**) Nanoparticles in zirconium matrix. (**c**) Typical energy dispersive spectrometer (EDS) results of precipitate.

The bright field TEM images of zirconium alloy before ion irradiation are shown in Figure 2. The size of zirconium crystal is above one micron. The insert image located at the top left in Figure 2a is a selected area electron diffraction (SAED) pattern of the zirconium matrix along the [1,2,3,4,5,6,7,8,9,10] crystal orientation. According to equilibrium phase diagram of Zr-Nb alloy, the thermally stable status of a binary zirconium alloy with a few percent of Nb is the solid solution of Nb in the α-Zr matrix when the temperature is below the monotectoid reaction temperature [31]. Niobium will precipitate as a β phase in the α-Zr grain matrix. According to TEM observation, the precipitates do not uniformly distribute in the zirconium matrix as illustrated by Figure 2a,b. The total volume fraction of the precipitates measured according to the bright field TEM images is about 4.0%, which is close to the value obtained by Nikulina [32], who measured the total volume fraction of precipitates at about 3~3.5% in Zr-1%Sn-1%Nb-0.4%Fe alloy.

Most of the precipitates have a roughly spherical shape with diameters mostly in the range of 30 to 60 nm although the diameters of a few precipitates are up to 100 nm. Quantitative analysis was conducted in a Climax Image Analysis system to obtain the roundness and sphericity of precipitates used to describe the precipitate shape. Roundness is a measurement of the length/width relationship, with values in the range 0–1.0. A perfect circle has a roundness of 1.0, while a needle shaped object has a roundness close to 0. Intuitively, the roundness is a comparison between the “strength” of the major axis and the “strength” of the minor axis. Roundness = 4 A/(πLL), where A is the area of the precipitate, and L is precipitate length. Sphericity is equal to 4 πA/P^2^, where P is perimeter of precipitate. According to bright field TEM images, the statistic results show that the roundness and sphericity of the precipitates are about 0.90 and 0.91, respectively. The measurement error for a precipitate is less than 3%. The count number for quantitative analysis is over 30. Therefore, the effect of slightly bending and tilting the sample on both the shape and the size of the precipitates is small.

The typical EDS results of the precipitate is shown in Figure 2c. Only zirconium peaks coming from the alloy matrix and niobium peaks coming from the nanoparticle can be observed in the EDS. Similar EDS results are obtained at several other nanoparticles. It can be concluded that most nanoparticles in the zirconium alloy matrix are β-Nb precipitates with a globular shape.

### 3.2. Precipitate Growth

**Figure 3:**
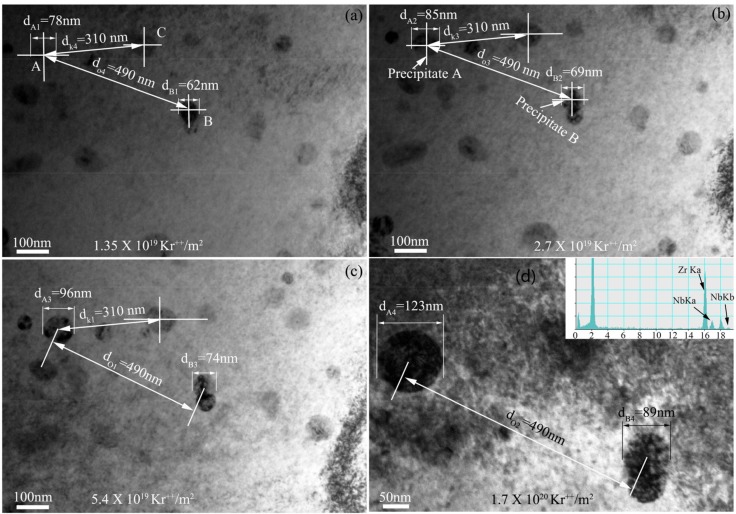
In situ TEM observation of nanoparticle evolution during 800 keV Kr^2+^ irradiation at 585 K with the ion fluence of (**a**) 1.35 × 10^19^ Kr^2+^/m^2^; (**b**) 2.7 × 10^19^ Kr^2+^/m^2^; (**c**) 5.4 × 10^19^ Kr^2+^/m^2^; (**d**) 1.7 × 10^20^ Kr^2+^/m^2^.

Irradiation of multi-component alloy by high energetic ions will lead to the change of phase microstructure such as the nucleation and growth of precipitates because of the enhancement of atomic mobility [26]. The morphology evolution of niobium nanoparticles with increasing of irradiation damage is shown in Figure 3. In the monitored area, the size of niobium particle is increased with increasing Kr^2+^ ion fluence. The size of Particle A is increased from 78 nm to 85 nm, 96 nm and 123 nm after irradiation with Kr^2+^ ion fluence from 1.35 × 10^19^ Kr^2+^/m^2^ to 2.7 × 10^19^ Kr^2+^/m^2^, 5.4 × 10^19^ Kr^2+^/m^2^, and 1.7 × 10^20^ Kr^2+^/m^2^, respectively. Obviously, the size of Precipitate A is increased with increasing Kr^2+^ ion fluence. Certainly, if the foil is hardly bent, the diffraction contrast may influence the observation of precipitates and further influence the size measurement of the precipitates. However, in the present work, the effect of diffraction contrast on size measurement was small when the foil was slightly bent during ion bombardment because the edge of Precipitate A is always sharp, as shown in Figure 3. Moreover, the roundness and sphericity of the precipitates are about 0.90 and 0.91, respectively, before ion irradiation according to the statistical results. The ion-irradiation-induced bend of foil also hardly changes the shape and size of the precipitates. In fact, from Figure 3a to Figure 3d, the distances between the center of Precipitate A and the center of Precipitate B are the same, d_o1_ = d_o2_ = d_o3_ = d_o4_ = 490 nm and the distances between the center of Precipitate A and the center of Precipitate C are also the same, d_k1_ = d_k3_ = d_k4_ = 310 nm, which indicates that the monitored area of the foil in the present work is not substantially more bent. Therefore, the size increment of the precipitate comes from the growth of precipitate induced by ion irradiation.

Like Precipitate A, the size of Precipitate B is increased from 62 nm to 69 nm, 74 nm, and 89 nm after irradiation with Kr^2+^ ion fluence from 1.35 × 10^19^ Kr^2+^/m^2^ to 2.7 × 10^19^ Kr^2+^/m^2^, 5.4 × 10^19^ Kr^2+^/m^2^, and 1.7 × 10^20^ Kr^2+^/m^2^, respectively. Our results are different from that reported in the reference. The size, distribution, and total concentration of the precipitates were almost kept constant before and after neutron irradiation [32]. The difference may be due to the different chemical composition and the incredibly different irradiation conditions. The chemical composition was Zr-1%Sn-1%Nb-0.4Fe, and their main experiment conditions were neutron irradiation up to 4.1 × 10^26^ nm^−2^ (*E* > 0.1 MeV) at 300 to 350 °C [32]. Wan [33] reported that the growth of precipitates was closely related to the atomic diffusion process. The irradiation-induced defects could accelerate the diffusion of solute atoms and promoted the growth of precipitates.

Ion irradiation causes alloying element redistribution. The irradiation-induced defects accelerate the movement of alloying atoms to the precipitates and makes them grow [34]. Nucleation will induce the change of free energy (∆*G*), including volume free energy (∆*G_V_*) and surface free energy (∆*G_S_*). When the crystal nucleus of fresh precipitate is formed at the previous precipitates in the zirconium alloy, the total change of free energy can be expressed as $\Delta G=\left(\frac{4}{3}\pi r^{3}\Delta G_{V}+4\pi r^{2}\sigma_{\alpha L}\right)\left(\frac{2-3cos\theta +cos^{3}\theta }{4}\right)$, where *r* is the radius of spherical crystal nucleus, and θ is the contact angle, ranging from 0 to 180°. If θ = 0°, the nucleation energy is equal to zero, which indicates that the precipitated Nb atoms freely occupy the lattice position of previous precipitates. Therefore, the nucleation energy of the precipitated Nb atoms attaching at previous precipitates is obviously lower than that of fresh precipitate formed independently in the zirconium matrix, which is the reason for precipitate growth.

The solid solution of elemental Nb is decreased in the zirconium alloy matrix with increasing Kr^2+^ ion fluence. The growth rate of Nb precipitates depends on the concentration of elemental Nb in the zirconium lattice and the irradiation conditions. The growth rate will increase first and then decrease because of the limited Nb content in the zirconium lattice. When the chemical potential gradient (∂μ/∂x) of elemental Nb in the precipitates and zirconium lattice is equal, the precipitates can stop growing. The chemical potential (μ_*i*_) is related with the Gibbs free energy of *i* atom, $\mu_{i}=\left(\frac{\partial G}{\partial n_{i}}\right)$. *n_i_* is the atom numbers of the *i* component. Ion irradiation will change the Gibbs free energy of elemental Nb in the zirconium alloy matrix and can induce the uphill diffusion of niobium. The diffusion coefficient of elemental Nb can be written as $D=kTB_{i}\left(1+\frac{\partial lnr_{i}}{\partial lnx_{i}}\right)$, where *B_i_* is the migration rate of elemental Nb; *T* is the temperature; *k* is the coefficient; *r_i_* is the activity coefficient; $x_{i}=\rho_{i}/\rho$. *ρ_i_* is the concentration of elemental Nb in the alloy matrix, which would be decreased by the ion irradiation. Although the irradiation experiment was carried out at 585 K, the growth of Nb precipitates may be mainly induced by Kr^2+^ ion irradiation and less by the thermal effect because no fresh precipitates can be detected after aging for 1500 h at 770 K [35].

**Figure 4:**
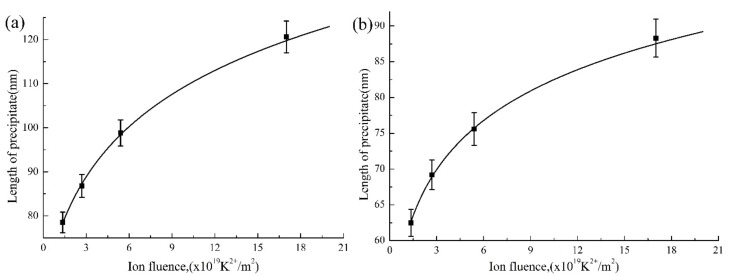
The relationship between the size of precipitate and Kr^2+^ ion fluence, (**a**) Precipitate A; (**b**) Precipitate B, as shown in Figure 3.

Quantitative measurement was conducted according to pixel resolution in a Photoshop software to obtain precipitate diameter at different ion irradiation conditions. The error value is less than 3%. The relationship between the size of the precipitate and Kr^2+^ ion fluence is shown in Figure 4. It can be seen that the size of the precipitate is increased with increasing Kr^2+^ ion fluence. The growth rate is large in the initial stage and will decrease to zero when the chemical potential gradient of elemental Nb in the precipitates and alloy matrix is equal.

### 3.3. Nanocrystallization

**Figure 5:**
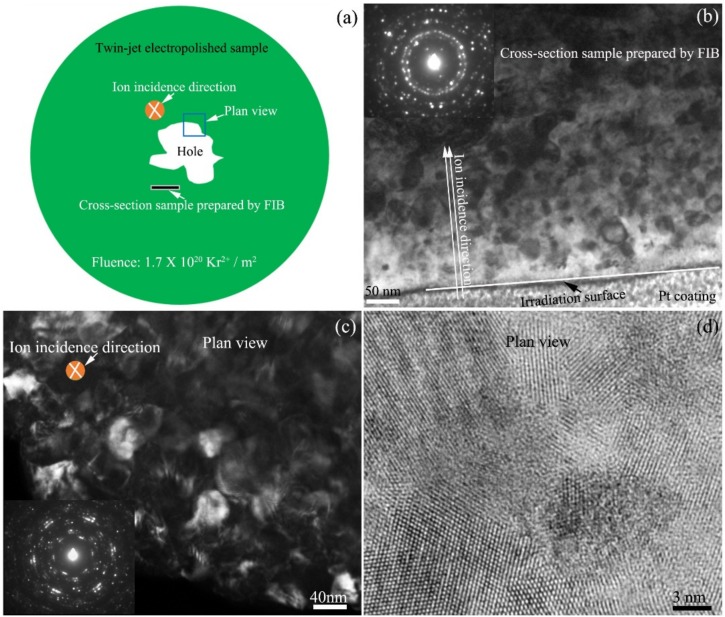
The microstructure of zirconium alloy irradiated by 800 keV Kr^2+^ ions with a fluence of 1.7 ×10 ^20^ Kr^2+^/m^2^. (**a**) The sketch of sample for in situ ion irradiation and the direction for microstructure observation; (**b**) the microstructure of the cross-section sample prepared by FIB; (**c**) the dark field TEM image showing the plan-view microstructure of the irradiated sample; (**d**) high resolution TEM image of (**c**).

The irradiation-induced dislocation walls will induce the formation of low-angle grain boundary in the microsize grain [36]. With increasing Kr^2+^ ion fluence, the zirconium grains with a micrometer size will be transformed into nanograins. Figure 5 shows the microstructure of zirconium alloy irradiated by 800 keV Kr^2+^ ion irradiation with a fluence of 1.7 × 10^20^ Kr^2+^/m^2^ at 585 K. Figure 5a indicates the sketch of the ion-irradiated TEM sample prepared by twin-jet electropolishing, the monitored region during Kr^2+^ ion irradiation, and the location of the FIB-prepared cross-section specimen in the ion-irradiated TEM sample after Kr^2+^ ion irradiation.

In order to observe the distribution characteristic of the microstructure along with the irradiation depth, the cross-sectional “TEM specimen” was prepared using the focused ion beam (FIB) “lift-out” technique covering a region that extended to areas far away from the irradiated surface as indicated by the white arrow in Figure 5a. The bright field TEM image of the cross-sectional microstructure of the irradiated sample is shown in Figure 5b. The direction of ion incidence, irradiated sample surface, and Pt coating are marked in a TEM image. The most severe problem is the damage layer produced as a result of the bombardment by highly accelerated gallium ion beams during FIB preparation [37]. The thickness of the damage layer with voids, dislocation, point defects, and amorphization is from several to tens of nanometers, which is decided by the preparation parameters such as the ion voltage and current, the ion incident angle, and the operation level of the experimenter. The damage layer induced by FIB will affect the quality of TEM images and interfere with the analysis. In order to decrease the thickness of the damage layer as far as possible, in the present work, a glancing angle of about 0.5° to 1°, a final beam current of 10 pA, and a beam voltage of 5 keV were used to clean the specimen surface, which significantly reduced the damage depth. The principal drawback of a 5 keV ion beam is that the etching rate and the resolution are lowered. The use of the 5 keV beam was be restricted to final milling. Meanwhile, in the present work, the beam voltage and current of Kr^2+^ ion irradiation in the IVEM-Tandem Facility are much higher than those of Ga^+^ ion during FIB preparation. Therefore, the effect of FIB damage on microstructural analysis can be neglected, and the analysis of the microstructure shown in Figure 5b is effective. The distribution band of nanocrystals under the irradiated surface can be seen. The width of the nanocrystal band (300 nm) is the same as the value calculated by SRIM software. The insert image located at the top right corner in Figure 5b is the SAED pattern of the zirconium matrix, which is typical nanocrystal diffraction pattern. Figure 5c is a dark field TEM image showing the plan-view microstructure of the irradiated sample, which has some characteristic microstructures with nanoscale. The insert image located at the left bottom in Figure 5c also shows nanocrystal diffraction rings. The high resolution TEM image of the plan-view microstructure is shown in Figure 5d.

Several theories were developed to describe phase-stability loss under irradiation [31,38,39,40]. The mechanism involves irradiation mixing, irradiation-enhanced diffusion, and irradiation-induced segregation. The irradiation-enhanced diffusion is controlled by the migration of non-equilibrium point defects (PD), vacancies and interstitial atoms due to ion irradiation. The irradiation-induced segregation is caused by the coupling of PD fluxes with the fluxes of alloying elements [31]. From in situ TEM observation, the zirconium grain with large amount of niobium particles was difficultly changed to Nano grain as shown in Figure 3. However, the zirconium grain without niobium nanoparticle was easily transferred to nanograin as show in Figure 5. This indicated that the Nb nanoparticles were the key factor that controlled the nanocrystallization of zirconium crystals. Nb particles are the sinks for point defects induced by Kr^2+^ ion irradiation. The interstitials and vacancies will move to Nb particles. The grain boundaries of the nanocrystals could also be the sinks to absorb defects, and the formation of more nanocrystals may slow down subsequent irradiation damage and improve radiation resistance of the materials [41]. Displacement damage drives complex microstructural and microchemical evolutions, including the accumulation of extended vacancy and SIA defect clusters, changes in the dislocation structures, nonequilibrium solute segregation, radiation-enhanced and-induced precipitation, and growing voids [42]. Xian-Ming Bai [41] reported that grain boundaries had a surprising “loading–unloading” effect. Upon irradiation, interstitials are loaded into the boundary, which then acts as a source, emitting interstitials to annihilate vacancies in the bulk. This unexpected recombination mechanism has a much lower energy barrier than conventional vacancy diffusion and is efficient for annihilating immobile vacancies in the nearby bulk, resulting in self-healing of the radiation-induced damage.

## 4. Conclusions

The microstructure evolution of the Zr-1.1Nb-1.51Fe-0.26Cu-0.72Ni zirconium alloy irradiated by 800 keV Kr^2+^ ions at 585 K was investigated by in situ transmission electron microscopy on the IVEM-Tandem Facility. The experiment results can be made as follows:

(1) Many β-Nb precipitates with a BCC structure are distributed in the as-received zirconium alloy with micrometer-size grains. Most of the precipitates have a globular shape. The roundness and sphericity of precipitates are about 0.90 and 0.91, respectively.
(2) Kr^2+^ ion irradiation induces the growth of β-Nb precipitates, which is due to the segregation of the dissolved niobium atoms in zirconium crystal structure and the migration to the existing precipitates. The size of precipitates is increased with increasing Kr^2+^ ion fluence.
(3) During Kr^2+^ iron irradiation, the zirconium crystals without Nb precipitates tend to transform to the nanocrystals, which is not observed in the zirconium crystals with Nb nanoparticles. The existing Nb nanoparticles are the key factor that constrains the nanocrystallization of zirconium crystals. The thickness of the formed Zr-nanocrystal layer is about 300 nm, which is consistent with the depth of Kr^2+^ iron irradiation.
